# A Randomized Controlled Trial of Increased Dose and Frequency of Albendazole with Standard Dose DEC for Treatment of *Wuchereria bancrofti* Microfilaremics in Odisha, India

**DOI:** 10.1371/journal.pntd.0003583

**Published:** 2015-03-17

**Authors:** Shantanu Kumar Kar, Bhagirathi Dwibedi, Anna Salomi Kerketa, Antaryami Maharana, Sudanshu S Panda, Prafulla Chandra Mohanty, John Horton, Cherubala P Ramachandran

**Affiliations:** 1 Regional Medical Research Centre (ICMR), Chandrasekharpur, Bhubaneswar, Odisha, India; 2 Department of Medicine, Kalinga Institute of Medical Sciences, Bhubaneswar, Odisha, India; 3 Ultrasound Clinic, Forest Park, Bhubaneswar, Odisha, India; 4 Tropical Projects, Hitchin, United Kingdom; 5 Universiti Sains Malaysia, Penang, Malaysia; Institute of Medical Microbiology, Immunology and Parasitology, GERMANY

## Abstract

Although current programmes to eliminate lymphatic filariasis have made significant progress it may be necessary to use different approaches to achieve the global goal, especially where compliance has been poor and ‘hot spots’ of continued infection exist. In the absence of alternative drugs, the use of higher or more frequent dosing with the existing drugs needs to be explored. We examined the effect of higher and/or more frequent dosing with albendazole with a fixed 300mg dose of diethylcarbamazine in a *Wuchereria bancrofti* endemic area in Odisha, India. Following screening, 104 consenting adults were randomly assigned to treatment with the standard regimen annually for 24 months (S1), or annually with increased dose (800mg albendazole)(H1) or with increased frequency (6 monthly) with either standard (S2) or increased (H2) dose. Pre-treatment microfilaria counts (GM) ranged from 348 to 459 mf/ml. Subjects were followed using microfilaria counts, OG4C3 antigen levels and ultrasound scanning for adult worm nests. Microfilarial counts tended to decrease more rapidly with higher or more frequent dosing at all time points. At 12 months, Mf clearance was marginally greater with the high dose regimens, while by 24 months, there was a trend to higher Mf clearance in the arm with increased frequency and 800mg of albendazole (76.9%) compared to other arms, (S1:64%, S2:69.2% & H1:73.1%). Although higher and/or more frequent dosing showed a trend towards a greater decline in antigenemia and clearance of “nests”, all regimens demonstrated the potential macrofilaricidal effect of the combination. The higher doses of albendazole did not result in a greater number or more severe side effects. The alternative regimens could be useful in the later stages of existing elimination programmes or achieving elimination more rapidly in areas where programmes have yet to start.

## Author Summary

In order to achieve global elimination of lymphatic filariasis, it may be necessary to consider alternative approaches to mass treatment using higher or more frequent dosing with the existing drugs used in the programmes. Outside Africa, the drugs used are albendazole and diethylcarbamazine given annually. The current study is the first to examine the use of higher or more frequent dosing in a bancroftian filariasis endemic area outside Africa. Four groups of infected subjects were followed for two years using standard techniques for monitoring drug efficacy in Odisha, India. We showed that higher doses of albendazole, or 6-monthly treatment, were more effective than the standard regimen in producing clearance of microfilaria and reductions in antigenemia. Additionally we found that these regimens were more effective in killing the adult filarial worms in the lymphatics. Importantly, the higher doses used did not increase the risk of side effects. These alternative approaches could accelerate lymphatic filariasis elimination either at the end of existing programmes or to permit new programmes to achieve their goals faster.

## Introduction

The Global Lymphatic Filariasis Elimination Programme (GPELF) had its inception in 1998 following agreements between the World Health Organization and the international pharmaceutical companies GlaxoSmithKline (GSK) and Merck to donate supplies of, respectively, albendazole and ivermectin for the programme [1]. By 2013 MDA programmes had commenced in 56 of the 73 endemic countries while 13 countries had progressed to the post-MDA surveillance phase. Nearly 984 million people were targeted and 4.4 billion doses had been distributed between 2002 and 2012 [2]. Although the programme envisaged 5–6 annual interventions, not all countries were able to start simultaneously, and in large countries, logistic problems have forced extension over many years. Despite steady progress over the past 15 years, there have been many obstacles [3]. Currently, several countries, mainly in Africa, have yet to start their programmes [4, 5]. Pockets of infection (Hot Spots) and less than anticipated decline in microfilaria rates because of poor treatment compliance have been observed especially in large countries in Asia where programmes have found it difficult to achieve the minimum recommended (65%) epidemiological coverage. These problems are likely to prolong the time to achieve LF elimination targets [6]. While these difficulties were foreseen at the onset, effective solutions still need to be worked out [7]. The global programme still relies on the original pharmaceutical tools (albendazole/ivermectin in Africa and albendazole/ diethylcarbamazine (DEC) elsewhere) and alternative treatments have yet to emerge [8]. Although development of macrofilaricidal compounds is advancing, these are still a long way from regulatory approval let alone widespread deployment [9, 10]. In such a scenario, improvement of current regimens could permit shorter periods of implementation, especially for those countries that are yet to start and bring greater effectiveness in dealing with hotspots or help accelerate sluggish programmes.

The initial drug regimens used at the initiation of the Global Programme were based on existing approved doses and did not require extensive additional investigation or drug approval. However no attempts to investigate the effectiveness of alternative regimens, either or use of higher or more frequent dosing were made [11]. A study conducted in Mali in a population that had received several cycles of ivermectin for onchocerciasis and several annual cycles of albendazole/ivermectin for lymphatic filariasis [12] showed that the higher dose of albendazole (800 mg) and ivermectin (200 mg) given twice yearly was more effective than the standard annual regimen. The effect of similar modifications of DEC containing regimens has yet to be investigated.

The current study was conducted in India to investigate the effect of increasing the dose of albendazole from 400 mg to 800 mg and /or increasing the frequency of dosing for *W*. *bancrofti* infection. Since the Indian programme uses a single dose of 300 mg DEC for all individuals > 15 years, the dose of DEC remained unchanged [13]. The study area around Bhubaneswar, in Odisha State, India has seen intermittent MDA activity initially using DEC alone and later DEC and albendazole.

## Materials and Methods

The study was conducted as an open level randomised clinical trial comparing effectiveness of four different regimens of DEC plus albendazole in individuals with *W*. *bancrofti* microfilaremia (S1 Fig). The study was registered with the ICMR Central Trial Registry-India, a participating centre in the WHO International Clinical Trial Registry Platform with number REF/2012/04/003501.

### Ethics Statement

The study was undertaken following ICH—GCP guidelines, with written informed consent from the participating individuals both for screening and prior to study enrolment. The protocol and consent information was approved by the Human Ethical Committee of the Regional Medical Research Centre, Bhubaneswar. The study protocol was further reviewed by Indian Council of Medical Research and Health Ministry Screening Committee, Govt. of India. The study was monitored by an independent external monitor (Dr T K Suma, Alleppey, Kerala), who checked completeness of records, compliance with GCP and accuracy of data entry.

### Conduct of Study

A *W*. *bancrofti* endemic area within thirty kilometres of Bhubaneswar in the Khurda district of Odisha was chosen for selection of subjects. Community meetings were arranged to provide information on the importance of the filariasis elimination programme and of the study prior to enrolling individuals for the study. In the filaria endemic villages, night blood was collected by finger prick between 19.00 and 23.00hrs from all individuals between 18 and 55 years of age during a door to door survey. Thick smears from 40μl blood were examined for microfilaria. Microfilaremic individuals who provided consent were further screened for eligibility. Subsequently, in eligible subjects, an intravenous blood sample (5ml) was collected between 21.00–22.00 hours and divided into two aliquots; 2ml with EDTA and 3ml without anticoagulant. From 1ml EDTA blood, the microfilaria count was determined using the Nuclepore filtration technique. Haemoglobin was estimated by automated cell counter (MS4, Melet Schloesing Laboratories); serum ALT and creatinine were measured by automatic biochemistry analyser (Cobas Integra 400, Roche) and pregnancy was excluded using a β-HCG rapid test on early morning urine samples. *W*. *bancrofti* (OG_4_C_3)_ antigen levels were also obtained using an ELISA kit (TropBio, Australia).

Subjects of either sex between 18 to 55 years of age, with microfilaria counts greater than 50/ml. of blood, who accepted the hospitalization and follow up requirements were included in the study. Female subjects who were either pregnant or lactating were excluded as were subjects with serum ALT level of > 30 units/dl, creatinine level > 1.2 mg/dl or haemoglobin level of <10 gm%.

Individuals satisfying the inclusion and exclusion criteria and providing a second consent for study inclusion were allocated sequentially to one of four treatment groups according to a pre-determined list generated in blocks of 26 using Prism software (Graphpad Prism 6.0).

Ultrasonography of inguinal, scrotal, axillary, thigh and arm lymphatic systems was performed prior to treatment using a Doppler Ultrasonography unit (GE, Logique 400 PRO, WIPRO) using a linear high resolution 7–12 Mega Hz probe, to record any “filarial dance” sign.

The individuals, in groups of 4–6, were then admitted to the hospital of the Kalinga Institute of Medical Sciences, Bhubaneswar, for study drug administration, observation and management of adverse events if required. The drugs were taken orally by the subjects around 8.30am after breakfast, under supervision of hospital staff. Where the subject vomited within 1hr of ingestion, the dose was repeated. The subjects were discharged from hospital after two days and followed at home for up to 7 days post drug intake.

A 2x2 factorial design with albendazole at two levels (400mg-low, 800mg-high) and number of doses per year at two levels (1dose-low, 2 doses-high) was used. The resulting four regimens are coded as S1, S2, H1 and H2. For each of the regimens 26 patients were allocated with a total of 104 for the study. These numbers were based on data comparing single and multi-dose regimens for the treatment of lymphatic filariasis [7, 14]. We assumed that standard annual therapy would clear microfilaremia in approximately 25% of subjects at 1 year; whereas the multi-dose therapy should give 75% clearance. Seventeen subjects per group are needed to detect this difference with two-sided alpha of 0.05 and 80% power while with 90% power it requires 23 per group. Allowing for 10% attrition, 26 subjects were recruited for each study arm.

The four treatment arms were as follows:

- S1: Diethylcarbamazine citrate (DEC) 300mg plus albendazole 400mg. oral single dose repeated annually.
- S2: DEC 300mg plus albendazole 400mg. oral single dose repeated six monthly.
- H1: DEC 300mg plus albendazole 800mg. oral single dose repeated annually.
- H2: DEC 300mg plus albendazole 400mg. oral single dose repeated six monthly.

The drug formulations used for the study were authorized drugs marketed in India as Banocide (DEC) 100mg tablets (GSK, Nashik, India) and Zentel (albendazole) 400mg tablets (GSK, Solan, India).

The subjects were followed up every 6 months up to 24 months for evaluation with drug administration as appropriate. Ultrasonography was repeated in those subjects with “filarial dance sign” detectable adult worm at base line, within 48–72 h of the first dose, and at 12 months and 24 months by the consultant ultrasonographer (C H Mohanty) who was unaware of the treatment received by the patients. Microfilarial count, OG_4_C_3_ antigen titre, urine β HCG test in females, serum ALT and creatinine were evaluated blind at follow up visits before the subjects received the next drug dose. All subjects were admitted to hospital for drug administration and for 2 days post drug to monitor for side effects / adverse events. Side effects/ adverse events were graded based on a previously used scale [15] and managed with simple remedies. Clinical evaluators were blind to the treatment given.

The clinical and laboratory information were recorded in a predesigned format. The data was entered into Excel and analysed using SPSS version 16 with appropriate tests for parametric and non parametric variables. Correctness of data was ensured by double entry and matching of entered data. The results were expressed as percentage changes and mean reductions at 6, 12 (the primary endpoint), 18 and 24 months. The chi-square test was used to compare the proportions, Student’s t-test for comparing mean differences between two regimens and analysis of variance for comparing all the four group means at 12 months, at 18 months and at 24 months. The net effect of the high levels was quantified using the contrast of a factorial design. The significance level was fixed at 5% level.

## Results

The study was initiated in October 2008 and final two year follow ups were conducted in September 2012, enrolling study subjects from 8 villages in the *W*. *bancrofti* endemic area of Khurda district in state of Odisha, India. This covered a rural area of around 150 square kilometres between Bhubaneswar (20.27^0^ N^,^ 85.84^0^ E) and Khurda (20.17^0^N, 85.67^0^E). The population is mostly middle and lower socioeconomic status within an agriculture based economy.

In all, 1751 individuals between 18 and 55 years of age were screened to identify 118 (6.74%) microfilaraemic subjects. 104 individuals who satisfied the inclusion/exclusion criteria and were willing to participate in the study were screened for eligibility. The subjects were randomly allocated to one of the 4 arms of the study and followed up with 6 monthly screening. All 104 individuals (26 in each of the four arms) received DEC + albendazole at baseline in the dosage prescribed for the respective arm. A 40-year-old male allocated to S1 arm died 9 months after the initial treatment following an acute abdominal emergency not considered to be related to the drug or study procedures. The remaining 103 study subjects all completed 24 months follow up.

The age and gender distribution of enrolled subjects (n = 104) was comparable among the four arms (*p* = 0.259 Student T test). There was a preponderance of males in all 4 treatment groups with approximately 17% females in each group, with most being below 35 years of age. Blood chemistry values were in the normal range in all subjects. The microfilarial density and mean OG_4_C_3_ units of the study population at enrolment are provided in Table 1. At baseline (0M), the geometric mean microfilarial density was not significantly different in the four groups and ranged from 348 to 459 mf/ml (*p* = 0.822). The mean OG_4_C_3_ antigen units were also comparable (*p* = 0.057). Ultrasonography prior to first dose detected adult worm nests with filarial dance sign (FDS) in 13 subjects in each of S1, H1 and H2 arms and 15 in S2 arm (p = 0.219).

**Table 1:** Baseline characteristics of the four populations in different treatment arms.

	S1	S2	H1	H2
**Age (Mean ± SD; IQR)**	33.7±11.6; 17.5	31.5±11.5; 18.8	28.2±10.5; 17.3	34.4±12.9; 22.0
**Mf Density (Mean ± SD)**	944.4±541.5	748.3±285.0	783.9±366.0	739.7±437.0
**Mf Density (GM)** *	457	348	386	459
**Og4C3 Antigen Units (Mean)** ^**#**^	12951.1	15834.2	13011.3	6514.3

* p = 0.822,

^#^ p = 0.057

The study population enrolled into each arm were compared in terms of age distribution, baseline Mf count and antigenemia. All the four groups were similar (p>0.05) in the baseline parameters.

**Fig 1:**
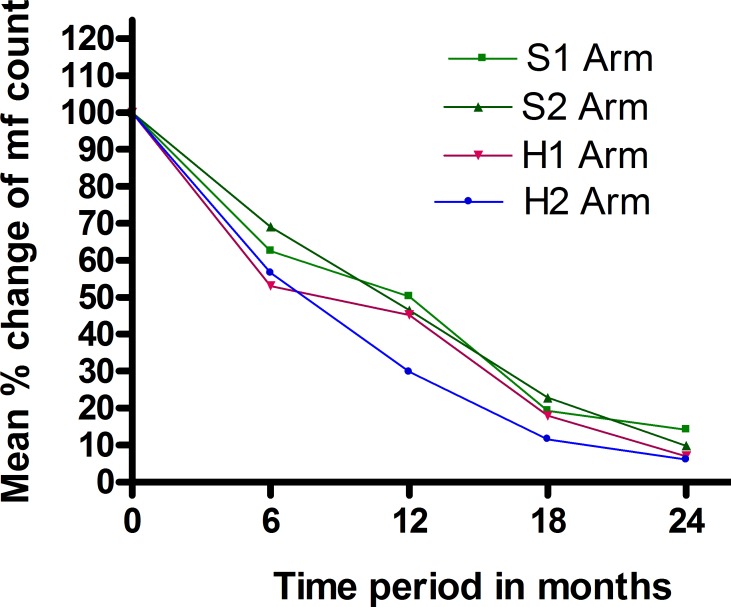
Mean percentage reduction in individual microfilarial count compared to baseline. Percentage changes from baseline values for individuals were calculated at each follow-up point and then means for each treatment group derived.

There was a progressive decrease in microfilaraemia and clearance of microfilaria from the circulation in all the four arms over the 24months (Table 2). The increase in dose and frequency reduced Mf counts significantly at the end of 12 months (*p* = 0.021) compared to the standard regimen (S1). At 24 months, Mf clearance was higher in H2 arm (76.9%) compared to other arms, (S1:64%, S2:69.2% & H1:73.1%) although this was not statistically significant (*p* = 0.77). The mean microfilarial density showed a sharp reduction by 6 months from baseline (S1:84.23%, S2:80.30%, H1:82.85%, H2:85.57%) in all four arms with a slower decline thereafter (Table 3). Mean percentage reductions in microfilarial density from individual baseline values are shown in Fig. 1. The differences in geometric mean Mf density are shown in Tables 4 & 5 (log transformed data). The analysis shows that, at 12 months, if albendazole is given biannually (S2 & H2), instead of annually (S1 and H1) there is an extra reduction in mf count of 0.5 log units and if albendazole is given 800 mg (H1 and H2), instead of 400mg (S1 & S2), there is an extra reduction of 0.94 log units. Biannual treatment with 800mg albendazole (H2) reduces the Mf count by 1.44 log units which is statistically significant (p = 0.021).

**Table 2:** Mf clearance at different follow up points in the four treatment arms.

Treatment arm	6 months	12 months	18 months	24 months
	Mf Neg. (%)	Mf Neg. (%)	Mf Neg. (%)	Mf Neg. (%)
**S1**	2/25 (7.7)	4/25 (16.0)	13/25 (52.0)	16/25 (64.0)
**S2**	1/26 (3.8)	5/26 (19.2)	12/26 (46.2)	18/26 (69.2)
**H1**	3/26 (11.5)	3/26 (11.5)	15/26 (57.7)	19/26 (73.1)
**H2**	3/26 (11.5)	7/26 (26.9)	17/26 (65.4)	20/26 (76.9)

Number of subjects completely cleared of microfilaria at the respective follow up point before the next dose is reflected in the table with Mf clearance as percentages. The table shows that the increased duration and higher dose (H2 arm) had higher Mf clearance. However the numbers are too low to demonstrate statistical significance, but there is a clear trend of extra reduction in microfilaremia in H2 arm.

**Table 3:** Percentage reduction in microfilarial density.

	S1	S2	H1	H2
**6 Months**	84.2	80.3	82.9	85.6
**12 Months**	92.0	95.2	93.0	97.3
**18 Months**	98.2	98.1	98.8	99.4
**24 Months**	99.1	99.4	99.8	99.8

Reduction in mf count in the individuals from each treatment arm from baseline values was calculated at the different follow up points and expressed as percentages. It shows sharp decline at 6 months follow up with gradual reduction thereafter. The difference between the arms is not significant.

**Table 4:** Change in microfilarial density in the treatment groups at different follow up points: Mean (SD) of Mf count/ml in blood for different treatment arms over 24 months.

Treatment arm	Baseline	6 months	12 months	18 months	24 months
S1	944 (1043)	149 (194)	75(92)	17 (53)	9 (22)
S2	748 (963)	147 (206)	36(40)	15 (33)	5(16)
H1	784 (880)	134 (336)	55 (90)	10(26)	1 (3)
H2	740 (737)	107(120)	20(43)	5 (10)	2 (5)

At 24 months, albendazole given biannually (S2 & H2), produced an extra reduction of 0.74 log units and if albendazole was given at the higher dose (800 mg) (H1 and H2) instead of 400mg, there was an extra reduction of 0.64 log units. Biannual treatment with 800mg (H2) albendazole reduced the Mf count by 1.09 log units which was statistically significant (p = 0.021). It can be concluded that an increase in dose and frequency can reduce Mf counts significantly at the end of 12 months and 24 months compared to the standard regimen (S1).

**Table 5:** Change in microfilarial density in the treatment groups at different follow up points: Mean (SD) of log (Mf) count for different treatment arms over 24 months.

Treatment arm	Baseline	6 months	12 months*	18 months	24 months
S1	6.12 (1.35)	4.12 (1.54)	3.75 (1.51)	2.39 (1.57)	2.34 (1.49)
S2	5.85 (1.29)	4.16 (1.54)	3.32 (1.09)	2.30 (1.50)	1.53 (1.60)
H1	5.95 (1.32)	3.47 (1.90)	2.88 (1.90)	2.41 (1.16)	1.25 (0.83)
H2	6.13 (1.07)	3.84 (1.77)	2.31 (1.50)	1.75 (1.53)	1.25.1.41)

*p = 0.028 Overall there is a significant difference between the treatment arms.

One way ANOVA was performed on the log transformed values to demonstrate any statistical significance in the effect of increasing dose or frequency of administration.

**Fig 2:**
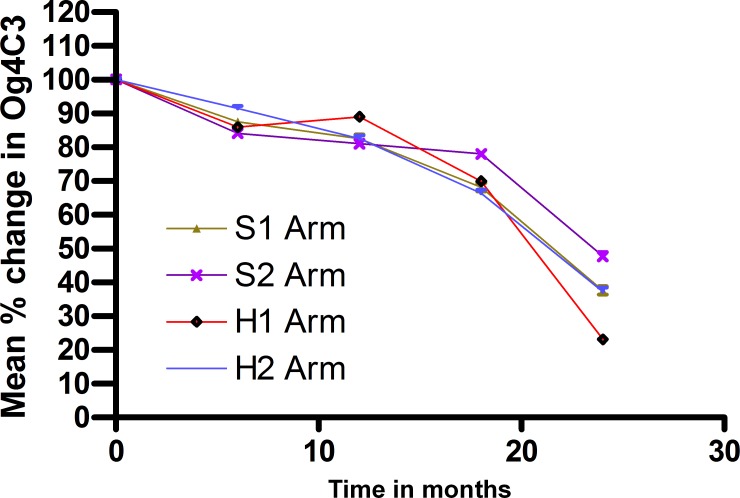
Mean percentage reduction on OG_4_C_3_ antigen levels compared to baseline. Percentage changes from baseline values for individuals were calculated at each follow-up point and then means for each treatment group derived.

Among the individuals showing FDS at baseline, repeat ultrasonography at day 3, 12 months & 24 months showed that disappearance of FDS was greater in H2 treatment arm, being complete at 12 months and 24 months compared to other 3 arms (Table 6), none of which showed complete clearance during the period of follow up. However these differences were not statistically significant due to the small numbers of individuals involved. Table 7 shows the change in antigen clearance and frequency of individuals showing reduction in antigen units at different time points (*p* = 12 M: 0.942, 18 M: 0.94 & 24 M: 0.23). Mean percentage reductions in OG_4_C_3_ from individual baseline values are shown in Fig. 2. OG_4_C_3_ antigen clearance was seen to start at 12 months in S1 & H2 arms and at 18 months in S2 and H1 arms and increased over time. At 24 months, OG_4_C_3_ clearance ranged from 26.9% to 73.1% (*p* = 0.007). Since the variance in the antigen units was very large due to wide range of values, logarithmic transformation was used to stabilize variance and one way ANOVA was performed on the log transformed values (Tables 8 raw data and 9 log transformed data)

**Table 6:** Change in adult worm prevalence on ultrasonography seen as Filarial Dance Sign (FDS).

Treatment arm	Baseline	Day 3	12 months	24 months
	Prevalence (n)	Prevalence (n)	Prevalence (n)	Prevalence (n)
**S1**	53.8% (14/26)	92.3% (12/13)	23.1% (3/13)	15.4% (2/13)
**S2**	57.6% (15/26)	86.7% (13/15)	6.7% (1/15)	6.7% (1/15)
**H1**	50.0% (13/26)	76.9% (10/13)	30.8% (4/13)	7.7%(1/13)
**H2**	50.0% (13/26)	61.5% (8/13)	0% (0/13%)	0% (0/13%)
*p value*	*>0*.*05*	*0*.*219*	*0*.*095*	*0*.*530*

**Table 7:** OG_4_C_3_ antigen status in 4 regimens group in different time points followed.

Treatment arm	6 Months		12 Months		18 Months		24 Months	
	No. showing reduction(%)	No. Cleared antigen(%)	No. showing reduction(%)	No. Cleared antigen (%)	No. showing reduction(%)	No. Cleared antigen(%)	No. showing reduction(%)	No. Cleared antigen(%)
**S1**	69.2% (18/26)	**0%**	72% (18/25)	**3.8%**	88% (22/25)	**(11.5%)**	100% (25/25)	**53.8%**
**S2**	76.9% (20/26)	**0%**	88% (23/26)	**0%**	80.7% (21/26)	**(7.7%)**	88% (23/26)	**26.9%**
**H1**	76.9% (20/26)	**0%**	73% (19/26)	**0%**	80.7% (21/26)	**(15.4%)**	96% (25/26)	**73.1%**
**H2**	61.5% (16/26)	**0%**	73% (19/26)	**3.8%**	80.7% (21/26)	**(15.4%)**	100% (26/26)	**61.5%**
**P values**	>0.05		0.942		0.942		0.234	

**Table 8:** Changes in antigenemia (OG_4_C_3_) over period in the four arms: Mean (SD) of (OG_4_C_3_) units for treatment arms over 24 months.

Treatment arm	Baseline	6 months	12 months	18 months	24 months
**S1**	12952(11101)	4600(5649)	6776(8132)	3958(6130)	1054(2776)
**S2**	15834(11724)	3791(4756)	3707(4619)	5643(7811)	2341(5869)
**H1**	13011(11002)	3889(3697)	5498(5458)	3376(5100)	195(813)
**H2**	6514 (5624)	4546 (4728)	3556(4818)	2686(5224)	726(2244)
***p* values**	0.057	0.9	0.18	0.18	0.017

**Table 9:** Changes in antigenemia (OG_4_C_3_) over period in the four arms:: Mean (SD) of log (OG4C) units for treatment over 24 months.

Treatment arm	Baseline	6 months	12 months*	18 months	24 months
**S1**	9.00 (1.07)	7.88 (1.04)	7.73 (2.13)	6.38 (2.73)	3.47 (2.79)
**S2**	9.19 (1.18)	7.74 (0.93)	7.45 (1.43)	7.16 (2.26)	4.37 (3.03)
**H1**	8.92 (1.24)	7.66 (1.29)	7.94 (1.41)	6.22 (2.47)	2.04 (2.41)
**H2**	8.40 (0.94	7.69 (1.44)	6.93 (2.11)	5.56 (2.63)	3.09 (2.57)

Results indicated, at 12 months (i) if albendazole is given biannually (S2 & H2), instead of annually (S1 & H1) there was an extra mean reduction of 0.66 log units in OG4C3 antigenemia (ii) if albendazole is given as 800 mg (H1 & H2), instead of 400mg (S1 & S2), there was an extra mean reduction 0.31 log units of OG4C3 antigenemia and (iii) if both are given at high level (H2) there is an extra reduction of 0.80 log units in OG4C3

At 24 months, (i) If albendazole is given biannually (S2 & H2), instead of annually (S1 & H1) there is an extra mean reduction of 0.53 log units in OG4C3 antigenemia (ii) If albendazole is given at 800 mg (H1 & H2), instead of 400mg (S1 & S2), there is an extra mean reduction of 0.86 log units of OG4C3 antigenemia and (iii) If both are given at high level (H2) there is an extra reduction of 1.38 log units in OG4C3 antigenemia. It can be concluded that increase in dose and frequency reduces OG4C3 log counts at the end of 12 months and 24 months.

Among individuals exhibiting adult worm (USG) at baseline, OG_4_C_3_ clearance was observed later at 18 month follow up (S1 = 7.7%, S2 = 6.6%, H1 = 7.6% & H2 = 15.3%) and increased over the subsequent 6 months (S1 = 53.8%, S2 = 26.6%, H1 = 76.9% & H2 = 53.8%).

Following drug administration, the frequency of adverse events was comparable in all four arms (S1: 68.38%, S2: 53.84%, H1:61.53% & H2: 50%) (*p* = 0.669) after the first dose (Table 10). The difference was also not significant at 6 months (*p* = 0.124) or 18 months (p = 0.305) dosing. However, at 12 months, S1 (28%) & H1 (15.4%) annual arms had a higher frequency of adverse events compared to S2 (3.8%) & H2 (3.8%) biannual arms. The adverse events noted were light-headedness (31.7%), fever (22.1%), body ache (4.8%), unsteadiness (4.8%), dizziness (2.8%), drowsiness (2.8%), fatigue (1.9%), chills (1.9%), itching (0.9%), and weakness (0.9%). There was no difference in the pattern of side effects noted in different arms (*p*>0.05). There were no severe drug related adverse events reported following any treatment, and the events that did occur could be managed conservatively.

**Table 10:** Number and percentages of subjects showing adverse events after administration of drugs in different treatment arms.

Treatment arm	0M	6M	12M	18M
**S1**	(17/26) 68.38%	0/0	7/25 (28%)	0/0
**S2**	14/26 (53.8%)	2/26 (7.6%)	1/26 (3.8%)	1/26 (3.8%)
**H1**	16/26 (61.53%)	0/0	4/26 (15.4%)	0/0
**H2**	13/26 (50%)	6/26 (23.0%)	1/26 (3.8%)	3/26 (11.5%)
***P***	0.665	0.124	0.027	0.305

## Discussion

The study was undertaken to investigate alternative regimens to those currently used in GPELF mass drug administration programmes outside Africa. This is the first attempt to rationalise or improve DEC intervention regimens aiming for greater effect on microfilaria clearance and /or adult worm suppression. Since new drugs are not available, the only alternative is either to increase doses of drugs or the frequency of dosing. Since DEC is already used at close to its acceptability ceiling by programmes (in India using 300 mg doses, rather than 6mg/kg), it is only possible to increase the albendazole dose [11]. Thus three test arms were used to compare with the standard regimen. This study enrolled adults infected with *W*. *bancrofti* having mf density >50mf/ml. Excluding individuals with a low mf count may reduce possible observer errors in counting low mf levels and also the interpretation of the impact of treatment on changes in mf density. Age, gender and endemicity matching of the population enrolled in different arms minimized the confounding effects.

The primary endpoint for the study was taken as Mf clearance at 12 months after initiation of treatment to be consistent with past studies, but follow up and analysis was continued for a further 12 months to establish whether any differences that might be seen at 12 months were sustained. Microfilarial clearance in all the four arms was progressive over time with a higher rate of decline in the mf prevalence in H2 arm. Although the difference in mf clearance between arms was not statistically significant over the 24 months of follow-up, the decline in mf prevalence in H2 arm gives the impression that if followed further, this arm could attain 100% mf clearance earlier than the other arms. This has the potential to shorten the period required for elimination. This was also indicated by the parallel reduction in microfilarial density where at both 12 and 24 months there was a significantly greater reduction in the high dose twice yearly arm (H2) compared to the other arms. A similar study using high dose twice yearly albendazole and ivermectin in Mali also showed enhanced suppression of microfilaria [12]. Although decline of antigen levels and rates of FDS occurred in all treatment groups, complete disappearance of FDS in worm nests by 12 months in the H2 arm without any recurrence at 24 months, and the decline in OG_4_C_3_ suggest that there is a significant macrofilaricidal effect at this dose.

Increased adverse events/side effects directly due to the higher dose of albendazole or as a result of interaction with DEC could be a limiting factor to deployment [16, 17, 18, 19]. However, 800mg albendazole has been extensively used for longer periods for systemic helminth infections such as echinococcosis and neurocysticercosis and is generally well tolerated [20]. In this study there was no increased frequency of adverse events when using 800 mg albendazole. All adverse events/side effects were self-limiting and no serious adverse events were encountered. The pattern of adverse events is typical of those encountered following treatment of microfilaria positive patients, and the higher frequency of adverse events encountered at 12 months by patients in the annual dose arms, whether high or low dose can be explained by the less effective clearance of microfilaria with an annual regimen.

The results of this study have important implications for GPELF in areas where onchocerciasis is not co-endemic. The annual low dose regimen performed well in this study and clearly remains the treatment of choice for the majority of situations. However, the availability of an alternative DEC-based regimen that uses a higher dose of albendazole with increased frequency could assist programmes to meet their elimination targets. In particular, this regimen may find use in areas/countries where MDA programmes are yet to be initiated, in “hot spots” identified during surveillance of ongoing programmes and in arresting transmission within shorter time frames [6]. However, programmes will undoubtedly need to weigh the benefits of a shorter duration regimen in the context of higher costs to programmes resulting from additional demands on logistics, training and community mobilization. Drug costs are likely to be restricted to procurement of DEC because of the commitment of GSK to provide albendazole free of cost to the programme. It is likely that programme implementation costs to the states/countries can be significantly curtailed by reducing the number of years required for elimination. Clearly, further studies with larger numbers to confirm and consolidate these data will be required before policy changes are made.

## Supporting Information

S1 Fig Consort flow diagram. (DOCX)

S1 Checklist Consort checklist. (DOC)
