# Supplementary material for: A Randomized Controlled Trial of Increased Dose and Frequency of Albendazole with Standard Dose DEC for Treatment of Wuchereria bancrofti Microfilaremics in Odisha, India
Source: PLoS Negl Trop Dis. 2015 Mar 17;9(3):e0003583. doi: 10.1371/journal.pntd.0003583 (PMC4363665; doi:10.1371/journal.pntd.0003583)
Supplement: S1 Fig — (DOCX) [file pntd.0003583.s001.docx]

**Supplementary Figure S1: Consort Flow diagram**

**CONSORT Flow Diagram**

## Enrollment

Pre eligibility screening : Door to door survey for identifying microfilariaemics (n=1716)

Analysed at 6,12,18,24 months (n=26)
♦ Excluded from analysis (n=0 )

Analysed at 6,12,18,24 months (n=26)
♦ Excluded from analysis (n=0)

Analysed at 6,12,18,24 months (n=26)
♦ Excluded from analysis (n=0 )

## Analysis

Analysed at 6 month (n=26)

At 12, 18, 24 months (n=25)
♦ Excluded from analysis (Lost to follow up) (n= 1)

Lost to follow-up (n=0) Discontinued intervention (n= 0 )

Lost to follow-up (n= 0 ) Discontinued intervention (n=0 )

Lost to follow-up (n=0 ) Discontinued intervention (n=0 )

Lost to follow-up at 12, 18 & 24M (n= 1 ) Discontinued intervention (n=0 )

Allocated to intervention (n= 26)

♦ Received allocated intervention (n=26)

Allocated to intervention (n=26)

♦ Received allocated intervention (n=26)

Allocated to intervention (n= 26)

♦ Received allocated intervention (n=26)

Allocated to intervention (n= 26)

♦ Received allocated intervention (n=26 )

Excluded (n= 14)

♦  Declined to participate (n= 14)

Assessed for eligibility (n=118)

**S1 ARM**

**S2 ARM**

**H2 ARM**

**H1 ARM**

**Randomized (n=104)**

## Allocation

## Follow-Up (6, 12, 18 & 24 Months)
